# eggNOG 5.0: a hierarchical, functionally and phylogenetically annotated orthology resource based on 5090 organisms and 2502 viruses

**DOI:** 10.1093/nar/gky1085

**Published:** 2018-11-12

**Authors:** Jaime Huerta-Cepas, Damian Szklarczyk, Davide Heller, Ana Hernández-Plaza, Sofia K Forslund, Helen Cook, Daniel R Mende, Ivica Letunic, Thomas Rattei, Lars J Jensen, Christian von Mering, Peer Bork

**Affiliations:** 1 Structural and Computational Biology Unit, European Molecular Biology Laboratory, Heidelberg, Germany; 2 Centro de Biotecnología y Genómica de Plantas, Universidad Politécnica de Madrid (UPM) – Instituto Nacional de Investigación y Tecnología Agraria y Alimentaria (INIA), Madrid, Spain; 3 Institute of Molecular Life Sciences and Swiss Institute of Bioinformatics, University of Zurich, 8057 Zurich, Switzerland; 4 Experimental and Clinical Research Center, a cooperation of Charité-Universitätsmedizin Berlin and Max Delbruck Center for Molecular Medicine, 13125 Berlin, Germany; 5 The Novo Nordisk Foundation Center for Protein Research, Faculty of Health and Medical Sciences, University of Copenhagen, Copenhagen N 2200, Denmark; 6 Daniel K. Inouye Center for Microbial Oceanography: Research and Education (C-MORE), University of Hawaii, Honolulu, HI 96822, USA; 7 Biobyte solutions GmbH, Bothestr 142, 69126 Heidelberg, Germany; 8 CUBE-Division of Computational Systems Biology, Department of Microbiology and Ecosystem Science, University of Vienna, Vienna 1090, Austria; 9 Germany Molecular Medicine Partnership Unit (MMPU), University Hospital Heidelberg and European Molecular Biology Laboratory, Heidelberg, Germany; 10 Max Delbrück Centre for Molecular Medicine, Berlin, Germany; 11 Department of Bioinformatics, Biocenter University of Würzburg, Würzburg, Germany

## Abstract

eggNOG is a public database of orthology relationships, gene evolutionary histories and functional annotations. Here, we present version 5.0, featuring a major update of the underlying genome sets, which have been expanded to 4445 representative bacteria and 168 archaea derived from 25 038 genomes, as well as 477 eukaryotic organisms and 2502 viral proteomes that were selected for diversity and filtered by genome quality. In total, 4.4M orthologous groups (OGs) distributed across 379 taxonomic levels were computed together with their associated sequence alignments, phylogenies, HMM models and functional descriptors. Precomputed evolutionary analysis provides fine-grained resolution of duplication/speciation events within each OG. Our benchmarks show that, despite doubling the amount of genomes, the quality of orthology assignments and functional annotations (80% coverage) has persisted without significant changes across this update. Finally, we improved eggNOG online services for fast functional annotation and orthology prediction of custom genomics or metagenomics datasets. All precomputed data are publicly available for downloading or via API queries at http://eggnog.embl.de

## INTRODUCTION

Identifying orthologs, those sequences diverging from a common ancestry after a speciation event, constitutes a fundamental task in molecular and evolutionary biology. Compared to paralogs, which are sequences diverged after a duplication event, orthologs are more prone to retain their ancestral function (1,2), even at long evolutionary timescales (3). Therefore, differentiating between these two subtypes of homology relationships is crucial to produce accurate functional predictions (2,4,5). It is also essential for proper analysis in, for example, phylogenetics and comparative genomics (6) or the study of cell-type evolution (7). Hence, several databases have been developed over the years that provide precomputed orthology predictions using different approaches and operational definitions (8–13). Most of those resources, including eggNOG, are part of the international consortium Quest for Orthologs (14), were standardized benchmarking approaches (15) and reference datasets are developed and shared.

eggNOG (evolutionary genealogy of genes: Non-supervised Orthologous Groups) is a public resource in which thousands of genomes are analyzed at once to establish orthology relationships between all their genes. Compared to similar databases, eggNOG focuses on providing: (i) comprehensive functional annotations for the inferred orthologs, (ii) predictions across thousands of genomes covering the three domains of life and viruses, and iii) hierarchical resolution of orthology assignments and fine-grained relationships (i.e. in-paralogies) based on phylogenetic analysis. For that, a species-aware clustering algorithm based on the concept of triangulation of best reciprocal hits (16) is applied to identify Orthologous Groups (OGs): sets of homologous sequences that started diverging from the same speciation event. As orthology relationships vary depending on the assumed reference speciation event (outgroup)—with increasing resolution toward the tips of the tree of life—since its inception in 2008 (17), eggNOG computes orthology predictions at different taxonomic levels. All OGs from all taxonomic levels are then functionally annotated and analyzed using phylogenetic methods, which allows users to further explore the history of speciation and duplication events within each OG, infer pairwise orthology relationships between specific species, or trace functional changes therein.

Here, we describe eggNOG v5.0, including the following improvements over previous versions: (i) a major upgrade of the underlying databases, featuring one of the most comprehensive selection of prokaryotic, eukaryotic and viral genomes available; (ii) updates in the online service for custom (meta-)genome annotation, now including options for fast orthology prediction and improved computational power via cloud computing and (iii) better visualization options of OGs and their associated functional data.

## UPDATES AND ADDITIONS SINCE PREVIOUS RELEASE

### Genomes update

eggNOG 5.0 has increased the number of genomes used for inferring orthology from 2031 core organisms to 5090. Viral proteomes have also been upgraded, increasing from 352 to 2502 proteomes collected from Uniprot and filtered by completeness (those with less than three proteins after in silico cleaving of polyproteins were discarded). In order to select best representative prokaryotic genomes, we used the SpecI species delineation method (18) against a total set of 25 038 genomes retrieved from RefSeq (19), obtaining 4445 reference species. Similarly, 477 eukaryotic genomes were collected from Ensembl (11) and other project-oriented resources (see online methods at http://eggnog.embl.de/). In all cases, genomes and proteomes were standardized and checked for completeness and minimum quality before inclusion into the database. For instance, incomplete prokaryotic genomes missing more than 4 out of 40 universal, single copy, marker genes (20) were excluded, as well as genomes that could not be assembled to fewer than 300 contigs or genomes with an N50 of <10 000.

### Taxonomic levels and non-supervised Orthologous Groups

An Orthologous Group (OG) is defined as a cluster of three or more homologous sequences that diverge from the same speciation event (16,17). Different OGs could therefore be inferred depending on the speciation split considered, that is, implicitly, the taxonomic resolution one considers. Older speciation events lead to larger OGs with more in-paralogs (duplication events occurred after the speciation) and higher functional divergence among their members. By contrast, recent speciations lead to smaller and usually more functionally specific sets of orthologs. For example, this implies that vertebrate-specific OGs would yield more fine-grained functional differentiation than OGs built using all eukaryotic species.

**Figure 1.**
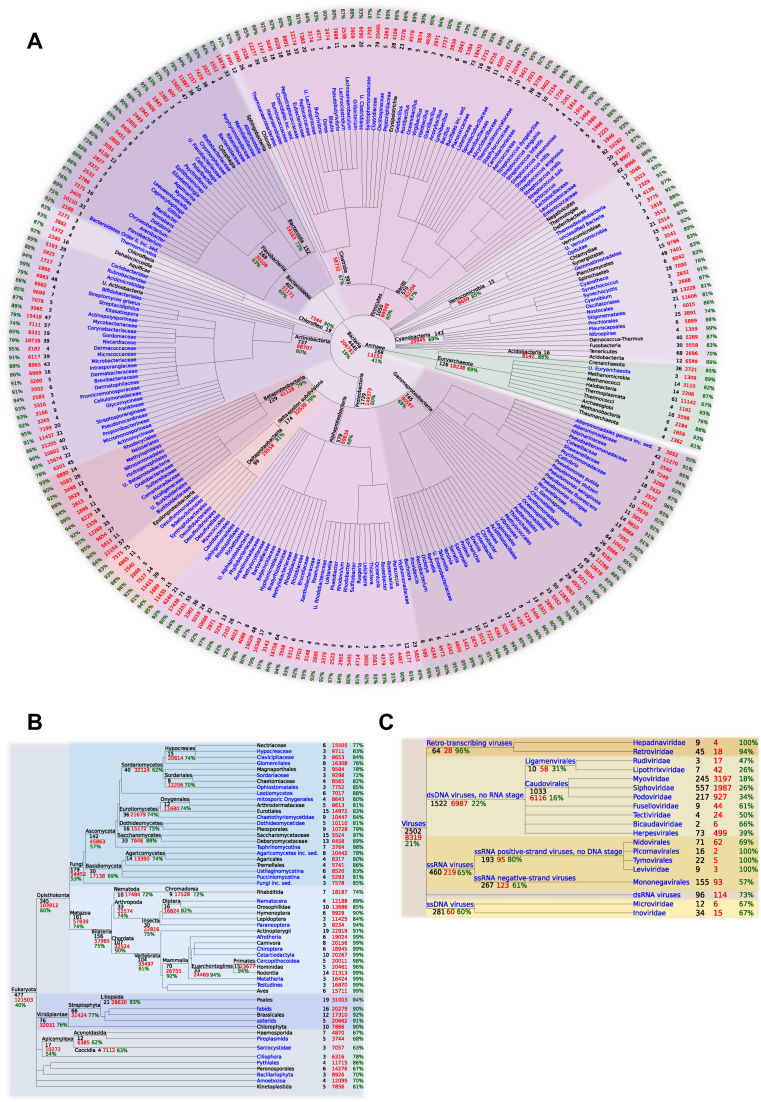
Taxonomic levels for which OGs have been independently computed based on (**A**) prokaryotic, (**B**) eukaryotic and (**C**) viral genomes. Names in blue indicate new taxonomic levels with respect to previous eggNOG versions. Numbers indicate the the amount of OGs per level (red), number of species covered (black) and functional annotation coverage (green).

In order to better reflect this taxonomic range and improve the precision of eggNOG functional predictions, in this version we have largely increased the number of pre-defined taxonomic levels (speciation splits) for which OGs are independently computed. In total, we applied the non-supervised eggNOG clustering method described in Jensen *et al.* (17) on 379 taxonomic levels, leading to 4.4M OGs (compared to 107 levels and 1.9M OGs in the previous version (21)). OGs were built using best reciprocal hits information derived from an all-against-all Smith-Waterman matrix provided by the SIMAP project (22). In addition, manually curated OGs available for the three domains of life were integrated into the corresponding levels in eggNOG, namely bacterial subset of COGs (23), archaeal arCOGs (24) and eukaryotic KOGs (25). Similarly, viral OGs were updated using deeper taxonomic categories, now descending to the family level. The taxonomic distribution in eggNOG v5.0, as well as the number of organisms, OGs inferred, and functional annotation coverage per level is shown in Figure 1.

### Hierarchical consistency of OGs

Relationships between more rootward OGs and their nested children OGs at more specific taxonomic levels were explicitly tracked and ascertained to be consistent, with exceptions only for mosic proteins with multi-domain combinations, where individual domains might have evolved independently (26,27). Hierarchical inconsistencies are the inevitable product of executing eggNOG’s clustering algorithm independently at each taxonomic level. Given that the set of species vary at each level, nested OGs might describe slightly incompatible evolutionarily histories for the same set of proteins. Solving those cases is particularly important for third-party applications (e.g. STRING (28)), in which information needs to be propagated across the hierarchy of taxonomic levels. Therefore, from version 4.5, we apply a post-processing step to ensure hierarchical consistency of all nested OGs.

In this database update, we have improved our methodology by implementing a more accurate strategy based on gene-tree reconciliation. Briefly, for each hierarchical inconsistency found, we subsample the proteins spanning the affected OGs and perform gene-tree to species-tree reconciliation. Each reconciled tree sample represents a vote towards one of the conflicting evolutionary hypotheses. We combine the reconciliations by majority voting to decide how to resolve the inconsistency. Given the large number of species in this version of eggNOG, we have however retained some size control heuristics, such as the rule that COGs should not be merged. A full description of the reconciliation method is available at https://github.com/meringlab/og_consistency_pipeline.

### Phylogenetics analysis

As in previous releases, all OGs in eggNOG v5.0 were analyzed using a comprehensive phylogenetic approach. Based on recent benchmarks (29), we adapted our phylogenomic strategy to the following steps: multiple sequence alignments inferred with Clustal Omega (30), soft alignment trimming by removing columns with less than five aligned residues, model testing using ModelFinder (31), maximum likelihood trees computed with IqTree (32) and branch supports calculated using the ultrafast bootstrap method (33). The full workflow was executed using the ETE toolkit v3.1.1 (34), which integrates the complete pipeline as a built in gene-tree workflow (code name ‘eggnog50_full’). For ∼57 000 OGs, due to the increasing gene family sizes, computation was not possible in this pipeline, so a fall-back method was used where IqTree was executed with the less-sensitive option ‘–fast’. All 4.4M trees were analyzed to infer speciation and duplication events (i.e. in-paralogy relationships) using the species overlap algorithm described in (35), leading to pairwise orthology tables (differentiating one-to-one versus many-to-many relationships) for each OG.

### Functional annotations

**Figure 2.**
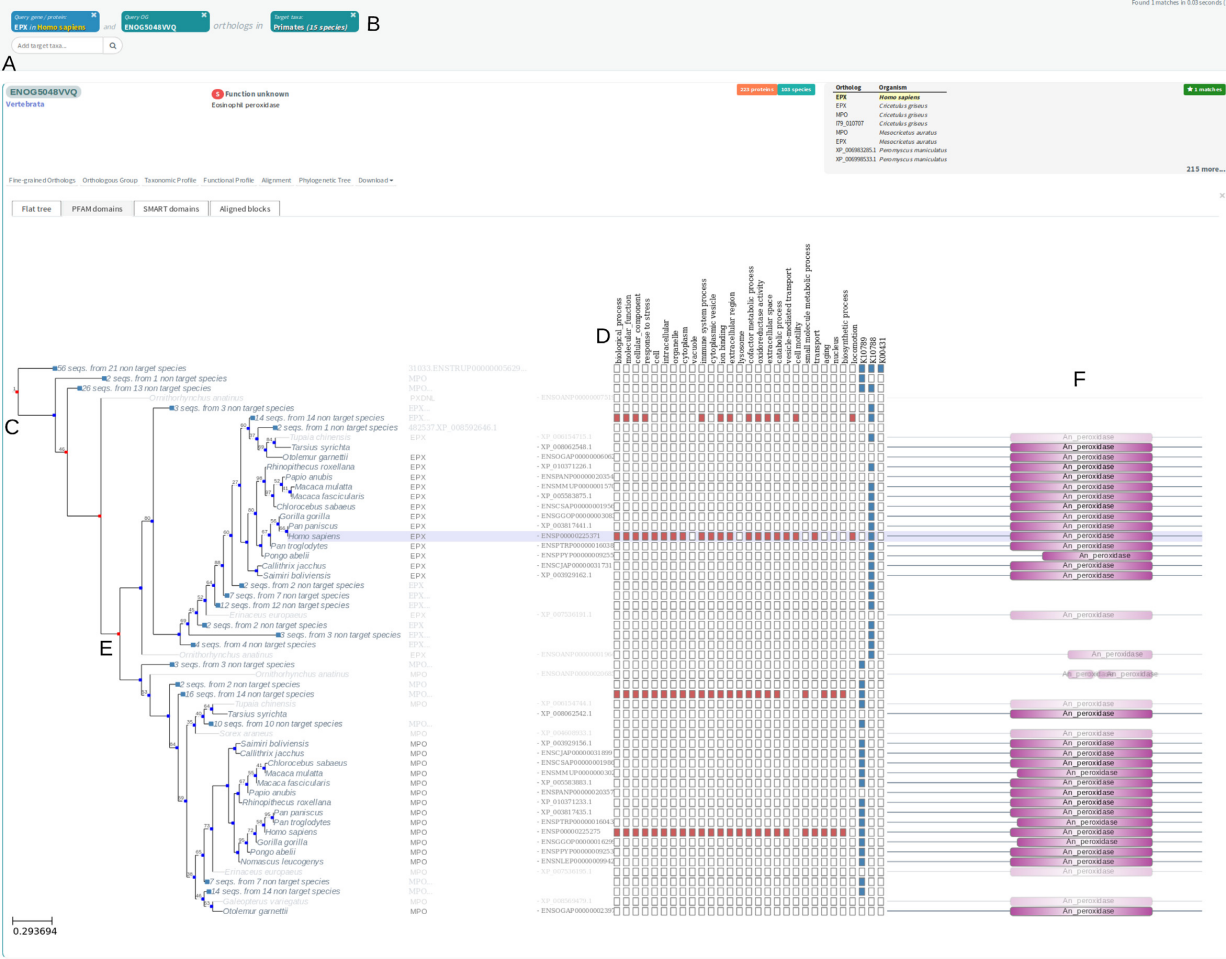
Visualization of the phylogeny associated to the OG ENOG5048VVQ at the vertebrate level (**A**) extracted from the eggNOG website. Target orthologs were restricted to primates in the phylogenetic tree to facilitate exploration (**B**). Duplication nodes (in-paralogies) are labeled in red, and speciation events in blue (**C**). The functional profile of each orthologous sequence is shown in the presence/absence matrix (**D**). Functional differences can be noticed at both sides of the duplication event separating EPX from MPO sequences (**E**) in both GO Slim terms (red squares in matrix D) and KEGG Modules (blue squares in matrix D), while having similar domain architectures (**F**).

Orthologous Groups were functionally annotated using updated versions of Gene Ontology (36), KEGG pathways (37), SMART/PFAM domains (38) and expanded to CAZy (39) and KEGG modules. Moreover, general free text descriptions and COG functional categories were updated for each OG using the automated text-mining and machine learning-based pipeline described in (21). In short, OGs were assigned text descriptions based on a heuristic to find the most informative text substring from either names of assigned SMART domains, assigned Gene Ontology terms, or common substrings in free text annotations from the source gene databases. In total, 80% of all OGs were annotated using at least one functional source. Finally, we improved the online visualization of functional annotations, which can now be explored from an evolutionary point of view by plotting functional descriptors together with the phylogenetic tree and the duplication/speciation events inferred for each OG (Figure 2).

### Fast functional and orthology assignments for custom user data

eggNOG v5.0 has also improved the underlying precomputed data used by the online version of eggNOG-mapper (40), a tool for rapid annotation of custom (meta-)genomes. Moreover, our online services are now cloud-enabled, permitting intensive computations required by functional annotation of massive datasets to run on dedicated servers with hundreds of CPUs available. We have also introduced a new option for fast batch orthology assignments of custom sets of sequences, which allows users to assign orthology relationships between novel genes and all genomes represented in eggNOG.

## BENCHMARK

The average quality of orthology predictions and functional annotations was benchmarked in order to estimate the effect of adding novel genomes. Both orthobench2 (41) and the Quest For Orthologs (QFO) benchmark (15) were used. Compared to eggNOG v4.5, we improved the performance in the orthobench's Bilaterian (from 72.1% to 73.1% *F*-measure) and Gammaproteobacteria test (from 93.2% to 94.7% *F*-measure). On the other hand, the QFO benchmark allowed us to evaluate the performance of both OG-based predictions and fine-grained predictions. Results show a clear tradeoff in the precision-recall ratio depending on the strategy selected, which in turn reflects different use cases of orthology assignments. OG-based predictions produced results with high recall values, predicting more than twice the number of orthologous pairs with <10.6% drop in average Schlicker similarity compared to the benchmark average in the Enzyme Classification and Gene Onthology Conservation tests. This high recall pattern is in general preferred by probabilistic prediction methods such as interolog inference in the STRING database (28). By contrast, fine-grained predictions showed higher precision values, while maintaining a similar recall as the previous EggNOG versions, which is usually preferred for accurate functional transfers. In general, for the majority of QFO benchmark tests, the performance of eggNOG 5.0 was slightly better or stayed at the Pareto line compared to previous eggNOG version (detailed plots and results are available at http://orthology.benchmarkservice.org). Taken together, this indicates that the large increase of genomes had no major impact on the quality of the inferred orthologous groups, suggesting the eggNOG approach continues to scale well.

## CONCLUSIONS AND PERSPECTIVES

By further streamlining and modernizing the automated approach for the construction of eggNOG orthologous groups, as well as synchronizing with improved or newly developed source databases (e.g. proGenomes for the classification of high quality prokaryotic genomes, (42)), we have been able to more than double core genome coverage for eggNOG, including extensive expansion of viral gene families, largely without loss of quality of orthology reconstruction or functional annotation. Due to a supervised increase of pre-defined taxonomic levels as basis for OG calculation, we almost tripled to number of OGs to 4.4M. Version 5 of eggNOG should thus be a useful resource for ecological, evolutionary or medical -omics analysis, also serving as an entry point for fast functional annotation of newly sequenced genes, genomes and metagenomes. We are currently working on conceptual and algorithmic improvements to be able to continue to keep pace with a vastly expanding number of organisms and meta-genomes sequenced.
